# A qualitative study of pharmacists’ perceptions and awareness of homoeopathic medicines in Durban, South Africa

**DOI:** 10.4102/safp.v65i1.5698

**Published:** 2023-12-26

**Authors:** Nokuthula H. Mavela, Ingrid M.S. Couchman, Themba Mgwaba, Celenkosini T. Nxumalo

**Affiliations:** 1 Centre for Excellence in Learning and Teaching, Durban University of Technology, Durban, South Africa; 2 Department of Homoeopathy, Faculty of Health Sciences, Durban University of Technology, Durban, South Africa; 3 School of Built Environment and Developmental Studies, College of Humanities, University of KwaZulu-Natal, Durban, South Africa; 4 Academic Development Unit, Faculty of Health Sciences, Durban University of Technology, Durban, South Africa; 5 Discipline of Nursing, School of Nursing and Public Health, University of KwaZulu-Natal, Durban, South Africa

**Keywords:** alternative medicines, complementary medicines, homoeopathic medicines, pharmacists, complementary and alternative medicines, perceptions, self-reported awareness

## Abstract

**Background:**

There is growing interest in the demand for and use of homoeopathic medicines by the public; however, little is known about the perspectives of pharmacists regarding the use of these medicines, particularly in the South African private health context.

**Methods:**

A qualitative approach using an exploratory cross-sectional descriptive design was used. Data were collected from a purposive sample of 15 participants comprising pharmacy managers, pharmacists and pharmacy assistants from six different conveniently selected private pharmacy retail outlets. Data were collected using individual interviews utilising a semi-structured interview guide. An audiotape was used to record the data which were transcribed verbatim and analysed thematically, following Tech’s steps of data analysis. Ethical approval to conduct the study was obtained from the Durban University of Technology’s Institutional Research Ethics Committee.

**Results:**

The findings of this study revealed four superordinate themes related to pharmacists’ perceptions and self-reported awareness regarding homoeopathic medicines. These are (1) negative perceptions regarding homoeopathic medicines, (2) perceived benefits of homoeopathic medicines, (3) poor knowledge and awareness of homoeopathic medicines and (4) capacity development and curriculum aspects.

**Conclusion:**

The findings highlight the need for an educational intervention on homoeopathic medicines targeting pre-service and in-service pharmacy practitioners, to enable them to provide effective education regarding all types of medicines as the demand for homoeopathic medicines increases.

**Contribution:**

The study findings provide evidence to support advocacy for an educational intervention to improve awareness and knowledge of pharmacists to enable provision of effective health education for patients. More research, however, is required to inform the contents of this training intervention for pharmacists.

## Background

Complementary and alternative medicine (CAM) refers to a set of traditional practices and treatment interventions that may be used, together with biomedicine, to provide a complementary and comprehensive health-related treatment intervention.^1^ Complementary and alternative medicine may also be used as the preferred practice or intervention of choice for certain health-related conditions.^2^ Homoeopathy is a traditional European medical practice that involves curative approaches to healthcare using natural remedies in various formulations.^3^ Homoeopathy thus forms part of a broad category of alternative health interventions which are classified as CAM and includes African traditional healing and medicine, naturopathy and oriental medicine.^4^ Complementary and alternative medicine also includes alternative medical therapies such as chiropractic and osteopathic medicine.^5^

A review of research on health-seeking behaviours suggests that there is growing interest and demand for CAM, with an increasing number of homoeopathic medicine sections being noted in many pharmacy outlets worldwide.^6^ Empirical data further show that homoeopathy is increasingly becoming recognised as a primary health care choice among most adults, especially in developing countries like South Africa.^7^ Evidence suggests a growing interest in learning more and having access to homoeopathic treatment interventions. In developing countries like South Africa, homoeopathic medicines are readily available for over-the-counter sales in various retail outlets such as pharmacies, health shops, retail supermarkets and health spas.^8,9,10^ Homoeopathic treatment interventions, including medications, form part of the package of CAM approaches which are being used in all parts of the world.^11^ In South Africa, homoeopathic medicinal products are often available in the private sector, with growing interest in both urban and rural areas.

There is growing interest in and use of homoeopathic medicines together with biomedicines as adjunctive treatment remedies among the general population globally.^12^ Healthcare workers, particularly nurses, medical officers and pharmacists, thus need to be informed about these medicines. Research studies have reported on the potential interaction between homoeopathic medicines and allopathic medicines which may have detrimental outcomes for patients.^13,14^ In the low- to middle-income countries such as South Africa, nurses, medical officers and pharmacists are often responsible to the dispensing of drugs related to treatment of various health conditions; moreover these healthcare workers have the professional obligation of providing education related to various drug treatment interventions as part of ensuring best patient outcomes in the treatment process. In this regard, these healthcare workers ought to be knowledgeable to provide information on side effects, mechanisms of action, drug-drug and drug-food interactions in the comprehensive package of health information that must provided.

The knowledge of CAM, such as homoeopathic medicines and treatment approaches, is thus crucial for healthcare workers because it facilitates implementation of best practices in terms of prescription, dispensing and provision of comprehensive educational interventions for patients during the issu.^15^ Awareness on the part of all healthcare workers also has implications for the provision of holistic and comprehensive healthcare for patients, which, in turn, has implications for health outcomes and broader public health.^16^

In the broader medical health sector, doctors, nurses, pharmacists, clinical associates and emergency care workers are most involved in the provision of medicines to patients through prescription, dispensing and administering of medicines. However, the literature reveals that pharmacists are the main custodians for medicines in the general medical stream of healthcare.^17,18^ Studies on the knowledge and perceptions of pharmacists regarding homoeopathic medicines have revealed limitations in the practices and awareness of pharmacists which are attributed to general negative perceptions regarding these approaches and the lack of sufficient integration of such content in their health science curriculum.^6,19,20^ Research conducted globally has also revealed that most of these studies were often done among medical and pharmacy students.^21^ Subsequently, there is paucity of this data among qualified and practising pharmacists in both the public and private health sector.

In sub-Saharan Africa, particularly South Africa, there are limited data available concerning pharmacists’ perceptions and knowledge of homoeopathic medicines, particularly using qualitative research approaches.^21,22^ The present study thus aims to explore and describe the knowledge and perceptions of pharmacy staff regarding homoeopathic medicines in Durban, South Africa. It is suggested that pharmacy staff are the custodians of all types of medicines; therefore, the present study findings have implications for an educational intervention to potentially improve pharmacy practices related to homoeopathic medicines. Moreover, there is growing evidence of an increasing demand for the use of homoeopathic medicines,^23^ thus requiring relevant healthcare workers to be trained in this regard. The study findings also create an awareness of the current perceptions and perceived knowledge of all categories of pharmacy staff regarding homoeopathic medicines, providing baseline evidence on the need for training in this area.

## Rationale for study

Taking cognisance of the aforementioned factors, together with an awareness of the rise in demand for and use of homoeopathic medicines, pharmacists, both at private and public health facilities, must be knowledgeable about homoeopathic medicines. This will allow for effective treatment-related health education interventions in terms of all medicines, particularly homoeopathic medicines which have been shown to be in demand among individuals, as they are often accessible in private pharmacies as over-the-counter medications. The possession of accurate knowledge and perceptions by pharmacists is also crucial for disease management and mitigation of any potentially negative consequences in the context of any multimorbid conditions that may be present.

## Methodology

### Study design

The study employed a qualitative approach using an exploratory cross-sectional design which was contextual in nature to explore and describe the perceptions and self-reported awareness of private sector pharmacy staff regarding homoeopathic medicines in Durban, South Africa.

### Setting

The study took place onsite at various locations within the greater Durban area of KwaZulu-Natal province in the Republic of South Africa, which were accessible to the researcher. Data were collected at selected retail pharmacy outlets in the province from a sample of various categories of pharmacy staff, namely, pharmacy assistants, pharmacists and pharmacy managers. Participants were from middle- to high-income earning socioeconomic standing. The participants were a mixture of black African and Indian ethnic groups.

### Sampling and recruitment strategies

Participants were selected from the various pharmacies in the greater Durban area where over-the-counter homoeopathic medication is sold. Managers of the pharmacies were given a gate-keeper letter by one of the primary researchers of the study. This letter informed the pharmacy management of the intent and purpose of the study. The gate-keeper letter further outlined the proposed methodology of the study in relation to sampling, data collection, data management and dissemination of findings. The letter also included information on how ethical standards of conducting research were to be ensured. Upon approval and permission from pharmacy management, including interest in the participation of their staff, an information letter, which included the contact details of the researchers and details regarding the ethical clearance of the study, was handed to pharmacy management.

The management of each respective pharmacy identified pharmacy staff in the relevant departments of the pharmacy as potential participants. Convenience sampling technique was used in this regard. The convenience sample group included pharmacy staff within the vitamin and nutrition departments, over-the-counter and/or self-medication dispensary counter and alternative medications department respective to each pharmacy. The identified potential participants were then approached and presented with the gate-keeper letter and information sheet. Upon interest and verbal approval of participation, the potential participants were given an informed consent form to fill in and sign to signify their full participation (see Appendix 2). According to their availability, pharmacy management determined the number of participants able to participate in the study. The numbers thus varied from one pharmacy to another. A total of 15 participants subsequently formed part of the study and included pharmacists, pharmacy managers and pharmacy assistants.

### Data collection procedure

Following ethical approval to conduct the study, an initial call and follow-up meeting was made with the private sector pharmacy owner and/or manager to give a brief description of the potential study. Permission to conduct the study was then established with interested pharmacies. Upon finalisation, a selected date and time was then chosen by the respective pharmacy management and/or owner. The research was conducted on a chosen day after fulfilling all protocols. Data were subsequently collected from a total of six private sector pharmacies which were all located in urban areas. Individual, face-to-face interviews were used to collect data from participants using a semi-structured interview guide (see Appendix 1). The data collection instrument comprised a demographics section and a section related to specific questions exploring participants’ perceptions and awareness regarding homoeopathic medicines. An audiotape was used to record all interviews which were conducted in English. The duration of each interview was between 25 min and 35 min. All interviews were conducted in private rooms to ensure confidentiality. Participants’ anonymity was maintained by not mentioning participants’ names during the recording and the use of codes to identify participants and their respective pharmacy outlets. A pre-test was conducted with the data collection instrument using two participants to validate the questionnaire prior to the actual data collection. There were subsequently no changes made to the questionnaire and the results were not included in the main study. All collected data were stored in a password-protected folder which was only accessible to the researcher. Each participant was assigned a unique identifier to save each recording and accompanying transcript to ascertain anonymity.

### Data analysis

Initial data analysis was conducted concurrently with data collection and ceased once saturation was reached at the 12th participant; data were however collected until the 15th participant to ensure that no new findings emerged following data saturation. The audio-recorded data were transcribed verbatim and then analysed following Tech’s steps of data analysis.^24^ The analysis of data was conducted in collaboration with experts in qualitative data analysis to ensure trustworthiness of the findings. Data analysis was an iterative process which entailed constant reading and re-reading of the interview transcripts to compare these with the audio-recorded data, thus ensuring trustworthiness of the findings. To ensure that the entire process of facilitating trustworthiness is achieved, the criteria set out by Lincoln and Guba^25^ were followed. To ensure credibility in this study, all interviews were audio-recorded and transcribed verbatim and field notes were taken during the data collection process. The audio-recorded data and transcriptions were stored safely in a password-protected folder for future reference. Furthermore, following transcription of data, participants were provided with transcripts and field notes to confirm whether this was truly a reflection of the information that they provided during the interview. The authors also repeatedly listened to the audio-recorded data to ensure that the transcripts matched with it; this subsequently ensured confirmability. Details of the research methodology including design, setting, sampling, data collection and data analysis processes are all provided to ensure transferability.

### Ethical considerations

Ethical approval to conduct the study was obtained from the Durban University of Technology’s Institutional Ethics Committee (IREC) (No. REC 74/14). Approval to conduct the study was also obtained from the private pharmacy outlets. Informed consent was obtained verbally and followed up in writing using a consent form and accompanying information sheet on the nature of the study.

## Results

A total of 15 participants from six private sector pharmacy outlets formed part of the study. The study participants comprised pharmacists, pharmacy managers and pharmacy assistants who were male (*n* = 4) and female (*n* = 11). The demographic details of participants are outlined in detail in Table 1.

**TABLE 1:** Demographic profile of participants.

Participant	Age (years)	Gender	Professional category	Clinical experience (years)
Participant 1	44	Male	Pharmacist	22
Participant 2	35	Female	Pharmacist	10
Participant 3	31	Female	Pharmacist assistant	3
Participant 4	28	Male	Pharmacist	6
Participant 5	37	Female	Pharmacist	15
Participant 6	26	Female	Pharmacist assistant	4
Participant 7	33	Female	Pharmacist	9
Participant 8	38	Male	Pharmacist	12
Participant 9	45	Female	Pharmacy manager	20
Participant 10	29	Female	Pharmacist assistant	4
Participant 11	41	Female	Pharmacy manager	19
Participant 12	25	Female	Pharmacist	3
Participant 13	36	Male	Pharmacist	13
Participant 14	23	Female	Pharmacy assistant	1
Participant 15	40	Female	Pharmacist	15

Data analysis yielded four superordinate themes on participants’ knowledge and perceptions regarding alternative and complementary medicines in Durban, South Africa. These themes were (1) negative perceptions regarding homoeopathic medicines, which yielded three subordinate themes, (2) perceived benefits of homoeopathic medicines, which yielded three subordinate themes, (3) poor knowledge and awareness regarding homoeopathic medicines, which yielded two subordinate themes, and (4) capacity development and curriculum aspects, which did not yield a subordinate theme. Table 2 outlines the themes and subthemes emerging in relation to participants’ knowledge and perceptions regarding homoeopathic medicines.

**TABLE 2:** Summary of themes and subthemes.

Theme	Subtheme(s)
1. Negative perceptions regarding homoeopathic medicines	1.1. Lack of interest in homoeopathic medicines 1.2. Uncertainty about the safety and efficacy 1.3. High price index
2. Perceived benefits of homoeopathic medicines	2.1. Increasing demand for homoeopathic medicines 2.2. Higher profit resale margins 2.3. Effectiveness of homoeopathic medicines
3. Poor knowledge and awareness of homoeopathic medicines	3.1. Inadequate knowledge about homoeopathic medicines 3.2. Limited access to knowledge on homoeopathic medicines
4. Capacity development and curriculum aspects	

### Negative perceptions regarding homoeopathic medicines

The superordinate theme of negative perceptions regarding alternative medicines revealed three subordinate themes, namely, lack of interest in homoepathic medicines, uncertainty about the safety and efficacy of these medicines and high price index. The verbatim quotations of participants’ responses are provided to support the accompanying subthemes.

#### Lack of interest in homoeopathic medicines

Participants’ responses appeared to suggest that they were not generally keen to sell homoeopathic medicines in their retail pharmacy outlets and had no interest in learning more about the medicines, unless prompted by a demand from clients. In incidences where they might become interested, participants revealed that they would be directed by the customers’ knowledge regarding these medicines. This was supported by the following statements:

‘Education is good, there is no harm, but I wouldn’t force my staff to attend training on homeopathic medicines, it will be their own personal choice.’ (Participant 1)

‘I do not actually sell homoeopathic products myself it’s only when a customer knows what exactly they want and if we have it, we will sell it to them, only if they specifically ask for it.’ (Participant 3)

‘That depends on how much we know about the product, and, in our line of work, we are not here to sell homoeopathy, we are here to do the other medication, basically prescribed medication. Normally what happens is if we keep a product, we obviously train on a product and based on the training if it makes sense, then we will sell it.’ (Participant 2)

#### Uncertainty about the safety and efficacy

Participants in this study expressed a general reluctance to provide complementary medicines as pharmaceutical items for sale in their pharmacy outlets, because of a lack of information regarding the scientific validity of treatment options and a general lack of information from a personal perspective. This was supported by the following excerpts:

‘I have not obtained much knowledge about it, just a few basics from product information inserts, companies, etc. I think better compound knowledge like information from clinical trials is needed to validate it.’ (Participant 6)

‘I believe that I lack knowledge enough to make informed suggestions to patients regarding the use of homoeopathic medications. Due to lack of knowledge regarding interaction of homoeopathic medication with conventional medication, especially in patients with chronic conditions, I am reluctant to suggest the use of such homoeopathic products in people on medication [*acute/chronic*].’ (Participant 7)

‘Effective in certain conditions, but further studies should be done of these products.’ (Participant 6)

#### High price index

Participants in this study reported that alternative medicines were much more expensive to keep as a stock item, as their retail price was higher, especially in medicines that are unique and were popular at the same time. Participants further revealed that the issue of high pricing was also reported by clients who were the potential customers for these medicines. This was supported by the following statements:

‘I noticed it’s quite expensive, especially the good stuff, but we do have a few things and most of it is actually imported from overseas so, automatically, if you are looking at cost, it’s going to be expensive, like that.’ (Participant 2)

‘Most of these medicines that supposedly treat the conditions that normal medicines treat are usually high-priced, so keeping them comes at an added cost.’ (Participant 4)

‘The public seem to have some level of interest in them, but when they see the price, they often complain that it’s unaffordable.’ (Participant 1)

### Perceived benefits of complementary medicines

This superordinate theme yielded three subordinate themes, namely, increasing demand for alternative medicines, higher profit resale margins and effectiveness of alternative medicines.

#### Increasing demand for homoeopathic medicines

Participants in this study reported receiving increasing requests by customers to provide alternative medicines at their retail pharmacy outlets. Participants further cited how customers would be specific about the brands and types of alternative medicines that they wanted to be sold. This was supported by the following statements:

‘It is gaining popularity and more customers are asking for it, especially reputable popular brands. We also get some homoeopathic products from overseas and some customers believe it works effectively.’ (Participant 1)

‘I have noticed recently that there have been quite a few people asking about specific complementary medicines and these clients often report to have heard specific information about the effectiveness of certain of these medicines.’ (Participant 5)

‘I find more people talking about alternative medicines and coming to ask for them.’ (Participant 6)

#### Higher profit resale margins

Participants reported that the resale value of alternative medicines was higher compared to traditional medicines. Moreover, this could be more easily facilitated with alternative medicines that are generally popular, but difficult to source locally. This was supported by the following statements:

‘It’s extremely profitable, it’s actually over-priced, uuh, it’s extremely effective, and it’s very, very easily used, extremely effective.’ (Participant 9)

‘It’s easier to inflate the prices when selling the medicines because some are not easily found anyway so those are the benefits of keeping them in stock sometimes.’ (Participant 11)

‘I [*have*] acquaintances who tell me that they have made a lot of money due to the increasing popularity of these medicines.’ (Participant 1)

#### Effectiveness of homoeopathic medicines

Certain participants in this study reported that alternative medicines were effective in treating certain acute and chronic conditions as verbalised by the customer and personally witnessed as clinical improvement was noted when certain clients were treated using alternative medicines. These participants, however, indicated that more research was necessary to support and scientifically validate this effectiveness. This was supported by the following responses:

‘It is easily used, if the customer, if you explain it properly to the customer and also homoeopathic stuff is working on the cause of the problem, and it’s helping the body heal itself, it’s not like over-the-counter medication that’s just treating symptoms so it’s actually better and it’s safer.’ (Participant 3)

‘Umh, it’s effective and easily used especially for people that are on medication and things, it’s one of the only stuff we can recommend.’ (Participant 12)

### Poor knowledge and awareness of homoeopathic medicines

This superordinate theme only yielded two subordinate themes, namely, inadequate knowledge about CAMs and limited access to knowledge resources on alternative medicines.

**Inadequate knowledge about homoeopathic medicines:** Participants reported that they lacked awareness and sufficient knowledge about alternative medicines mainly because they had not been formally educated about these in their undergraduate studies. This was supported by the following statements:

‘I am uncertain of the need and also do not know much about homoeopathic medicines so I can’t say much about them because of my limited knowledge.’ (Participant 1)

‘I believe that I lack knowledge enough to make informed suggestions to patients regarding the use of homoeopathic medications. Due to lack of knowledge regarding interaction of homoeopathic medication with conventional medication, especially in patients with chronic conditions, I am reluctant to suggest the use of such homoeopathic products to people on medication [*acute/chronic*].’ (Participant 7)

‘I don’t know much about homoeopathic drugs you know, but I know it’s safe to use. That’s all I know.’ (Participant 10)

**Limited access to knowledge on homoeopathic medicines:** Participants’ responses revealed that they had limited access to sources of information about alternative medicines. These participants further revealed that this limited knowledge was mainly through searching for information online, and through the minimal in-service training from certain alternative medicine pharmaceutical companies, as such, this was limited to specific brands of products. This was supported by the following statements:

‘Having a B Pharmacy degree, I received only one presentation by a single company on homoeopathic medication. That was not information enough to sustain me for a lifetime of patient interactions.’ (Participant 7)

‘I don’t know much about homoeopathic you know, but I know it’s safe to use, yeah, that’s all I know.’ (Participant 10)

‘Okay, most of the time the reps normally train us, but what I also do, I normally google it if I am not sure about a product, because not everything we get trained on, mainly like [*certain homeopathic product*], like with the [*certain homeopathic product*] range I have to Google it.’ (Participant 3)

### Capacity development and curriculum aspects

This was a stand-alone superordinate theme, which was related to participants’ perceptions about the interventions necessary to improve the general awareness of pharmacists regarding CAMs. Participants revealed that they perceived such awareness to be imperative in the clinical practice of pharmacists. Alluding to this, participants stated that more research data and related pharmaceutical knowledge should be available through the inclusion of this content in the formal training curriculum of pharmacists. This was supported by the following statements:

‘I am uncertain of the need [*for training*] and also do not know much about homoeopathy, so I can’t say what exactly is vital and must be included but the basics, I guess: what it is and how it works and scientific research behind it as with most medicines.’ (Participant 11)

‘No, I think they need to be taught about what it is, because a lot of, like … even like … staff don’t know what it is. They just know the basics, like this is going to help with this, like, for example, [*certain homeopathic product*] they don’t really know like where it is from and what’s it all about, and they still need to get taught and trained.’ (Participant 3)

‘Umh, think it would be good to have that background knowledge when prescribing to, or advising customers, I think it would be good to have that background knowledge of how it all started.’ (Participant 4)

## Discussion

The aim of this study was to explore the perceptions and self-reported awareness of pharmacists regarding homoeopathic medicines at private sector retail outlets in Durban, South Africa. The data revealed negative perceptions in the form of a lack of interest and uncertainty regarding homoeopathic medicines. The findings also revealed the perceived benefits of these medicines which were related to greater effectiveness, increasing demand and higher profit resale margins. The findings also revealed that pharmacists working at private pharmacy outlets in Durban, South Africa, generally lack knowledge and awareness regarding homoeopathic medicines.

This study revealed that pharmacists in private retail pharmacy outlets harboured negative perceptions regarding homoeopathic medicines. The negative perceptions were found to be in the form of a lack of interest in the sale of these medicines at their retail outlets and a general uncertainly about the effectiveness of these medicines. Research studies on South African healthcare workers’ attitudes and perceptions regarding homoeopathic medicines suggest that while certain health professionals have positive attitudes towards homoeopathic medicines, they often have little knowledge regarding homoeopathic medicines, resulting in a lack of integration of these medicines into mainstream health practice.^26,27,28^

In the context of the present study, the lack of interest for homoeopathic medicines may be attributed to the lack of knowledge regarding these medicines, a finding which has been reported in earlier studies on South African healthcare workers’ knowledge, attitudes and perceptions regarding homoeopathic medicines.^26,27,28^

Negative perceptions about homoeopathic medicines also prevailed in relation to the high price index of these medicines. In this study, the issue of the high price index was reported to participants through the medium of customer complaints and was noted when attempting to keep a supply of the medicines at the respective local pharmacy outlets. This finding represents one of the potential barriers to the uptake of alternative medicines by community members and may also explain the lack of interest in alternative medicines, as reported earlier in this study. The issue of the generally high prices of medicines is a phenomenon that is also experienced in traditional pharmacological therapies, as it necessitates regulations regarding pricing of medicines to ensure universal access for all, in line with the sustainable development goals.^29,30^ In this regard, there should also be specific policies and protocols that govern the price index of homoeopathic medicines. In this regard, the South African Health Products Regulatory Authority has a role to play in relation to enforcing safety and pricing standards in relation to homoeopathic and other forms of CAMs.

This study also reported the perceived benefits of homoeopathic medicines as expressed by participants. In this regard, participants revealed perceived benefits of increasing demands for homoeopathic medicines, higher profit resale margins and effective homoeopathic medicines. The reported increasing demand for homoeopathic medicines is also consistent with the findings of studies which have revealed high rates of patient satisfaction regarding homoeopathic medicines and treatment approaches in the South African context.^31,32^

While there is evidence of reported satisfaction with the efficacy of homoeopathic medicines, rigorous testing and evaluation of these medicines is necessary, so that drug usage may be integrated into mainstream medical treatment and care. Similarly, Lin et al.^33^ not only noted the need for more evidence surrounding the safety and efficacy of complementary medicines but also cautioned about the methodological difficulties of conducting such evaluations.

Conversely, as established by Peter Bai et al.,^34^ there is an increasing demand and usage of these medicines which may also have a significant impact for mainstream medicines, having implications for pharmacy practice, particularly in relation to patient education, as pharmacists are the custodians of all types of medicines. This necessitates pharmacists being able to provide education concerning the dosage instructions, side effects, adverse effects and special medical precautions to be adhered to in an event where complementary medicines are requested. Scholars have argued that pharmacists and medical doctors have an ethical responsibility to patients to be knowledgeable about all forms of CAM, including homoeopathic medicines to ensure that patients are aware and protected from drug-herb interactions that may occur when using both biomedicine and CAMs.^35,36,37^ This is of relevance to pharmacists in private sector retail outlets where over-the-counter medications are easily accessible and are often requested.

The study also revealed poor self-reported knowledge about homoeopathic medicines which was participants’ perceived and self-reported awareness, which could be related to the lack of access to information which was reported by participants. Thandar et al.^38^ in their study to explore the knowledge, attitudes and practices towards the use of CAM among community pharmacists in Durban, South Africa, found that pharmacists demonstrated poor knowledge and communication about CAM highlighting the need for education programmes and inclusion in the undergraduate curriculum. The present study findings imply that information about alternative medicines, particularly homoeopathic medicines, is neither readily available nor widely distributed. This suggests that pharmacists may be unable to provide holistic and comprehensive information to customers and patients about drug-drug interactions as may be required by patients.

The findings of this study also revealed that participants perceived pre-service and in-service training regarding homoeopathic medicines as necessary. Moreover, the findings also suggested that the education of pharmacists regarding these medicines be underpinned by scholarly, informed evidence about these medicines that is founded upon biomedical empirical research methods.

## Limitations

The study was conducted in only six pharmacy retail outlets which were situated in Durban, KwaZulu-Natal; hence, the reported findings may not necessarily provide a holistic perspective on pharmacists in the area or in KwaZulu-Natal entirely. Moreover, the study sought to elicit self-perceived awareness and did not measure actual knowledge objectively. Consequently, these data indicate that while education is needed, it cannot determine the extent or scope of an educational intervention.

## Conclusion

The present study was conducted to explore the perceptions and awareness of pharmacy staff regarding homoeopathic medicines in Durban, South Africa. The findings highlight the need for an educational intervention on homoeopathic medicines targeting pre-service and in-service pharmacy practitioners, to enable them to provide effective education regarding all types of medicines as may be required by patients as the demand for homoeopathic medicines increases. More research is, however, required to explore the extent of knowledge of pharmacists regarding homoeopathic medicines. Quantitative research design is thus recommended in this instance and collaboration between homoeopathic professionals and pharmacists is required in designing a training intervention for pre- and in-service pharmacists.

## Figures and Tables

**FIGURE 1-A1:**
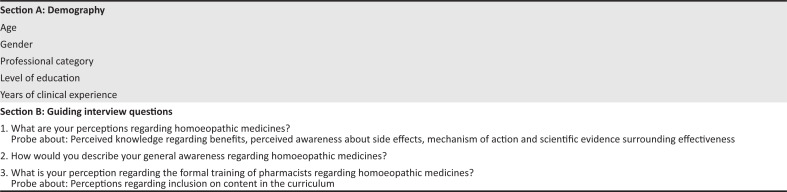
Data collection instrument.

## Appendix 2

### LETTER OF INFORMATION

**Title of the Research Study:**

‘**Perceptions and self-reported awareness of Homoeopathic medicines: A qualitative study of pharmacy staff in Durban, South Africa**.’

**Principal investigator(s)/researcher:**

**Co-investigator(s)/supervisor(s):**

**Brief Introduction and Purpose of the Study:** To explore and describe the perceptions and self-reported awareness of private sector retail pharmacy staff regarding homoeopathic medicines in Durban, South Africa.

**Outline of the Procedures:** Individual face-to-face interviews will be conducted using a semi-structured interview tool. An audiotape will be used to record all interviews but the name of the participant and all personal information will not be included in any recordings; thus confidentiality will be maintained even so in transcription. Interview will last for approximately 1 h.

You will be asked a series of questions concerning your perceptions and awareness of homoeopathic medicines. Participation in the study is voluntary, and you may withdraw from participating at any time without facing any penalty.

**Risks or Discomforts to the Participant:** There are no dangers to participating in this study at all.

**Benefits:** There will be no direct benefit to you for participating in the study; however, it may provide important baseline information that may inform the training needs for pharmacists with regard to homoeopathic medicines.

**Reason(s) why the Participant May Be Withdrawn from the Study:**

- You may at any instance ask questions or raise any concerns relating to the study.
- You have the right to withdraw from the study at any time. Please be aware that information you may have given prior to your withdrawal including recordings will be destroyed and omitted out of the study. There is no penalty for withdrawal from the study or implications as it is voluntary participation.
- If at any instance you feel uncomfortable with the voice recorder, you may ask that it be switched off and it will not be used.
- If there are any other concerns post the interview, you may kindly get in touch with the researcher and raise them.

**Remuneration:** There will be no monetary or other materials remuneration by the researcher for participation in this study. It is strictly voluntary.

**Costs of the Study:** There is no cost implication for the participants.

**Confidentiality:** The interview will be semi-structured; thus questions will be asked that will need your response, and these will be recorded using a voice recorder but the name of the participant and all personal information will not be included in any recordings, thus confidentiality will be maintained even so in transcription.

**Research-related Injury:** Due to the nature of the research, there are no foreseeable injuries or any related injuries.

**Persons to Contact in the Event of Any Problems or Queries:**

### CONSENT

Statement of Agreement to Participate in the Research Study: I hereby confirm that I have been informed by the researcher, ____________ (name of researcher), about the nature, conduct, benefits and risks of this study – Research Ethics Clearance Number: ___________.

I have also received, read and understood the above written information (Participant Letter of Information) regarding the study.

I am aware that the results of the study, including personal details regarding my sex, age, date of birth, initials and diagnosis, will be anonymously processed into a study report.

In view of the requirements of research, I agree that the data collected during this study can be processed in a computerised system by the researcher.

I may, at any stage, without prejudice, withdraw my consent and participation in the study.

I have had sufficient opportunity to ask questions and (of my own free will) declare myself prepared to participate in the study.

I understand that significant new findings developed during the course of this research which may relate to my participation will be made available to me.

____________________ __________ ______ _______________

Full Name of Participant Date Time Signature/Right Thumbprint

I, ______________ (name of researcher) herewith confirm that the above participant has been fully informed about the nature, conduct and risks of the above study.

_________________ __________ ___________________

Full Name of Researcher Date Signature

_________________ __________ ___________________

Full Name of Witness (If applicable) Date Signature

_________________ __________ ___________________

Full Name of Legal Guardian (If applicable) Date Signature

## References

[1] Pal SK. Complementary and alternative medicine: An overview. Curr Sci. 2002;82(5):518–524.

[2] Debas HT, Laxminarayan R, Straus SE. Complementary and alternative medicine, Europe PMC; 2011.

[3] Loudon I. A brief history of homeopathy. J R Soc Med. 2006;99(12):607–610. 10.1177/014107680609901206 17139061 PMC1676328

[4] Schmacke N, Müller V, Stamer M. What is it about homeopathy that patients value? And what can family medicine learn from this? Qual Prim Care. 2014;22(1):17–24. 24589147

[5] Walker BF, Armson A, Hodgetts C, et al. Knowledge, attitude, influences and use of complementary and alternative medicine (CAM) among chiropractic and nursing students. Chiropr Man Ther. 2017;25(1):1–8. 10.1186/s12998-017-0160-0 PMC5644107 29075438

[6] Ashraf M, Saeed H, Saleem Z, et al. A cross-sectional assessment of knowledge, attitudes and self-perceived effectiveness of complementary and alternative medicine among pharmacy and non-pharmacy university students. BMC Complement Altern Med. 2019;19(1):95. 10.1186/s12906-019-2503-y 31053114 PMC6500055

[7] Erwin K, Marks MM, Couchman IMS. Homeopathic health care in a low-income housing estate in Durban: Possibilities for a plural health care model in South Africa. Int J Health Wellness Soc. 2014;3(3):1–16. 10.18848/2156-8960/CGP/v03i03/41041

[8] Singh V, Raidoo DM, Harries CS. The prevalence, patterns of usage and people’s attitude towards complementary and alternative medicine (CAM) among the Indian community in Chatsworth, South Africa. BMC Complement Altern Med. 2004;4(1):1–7. 10.1186/1472-6882-4-3 15018622 PMC356921

[9] Peltzer K, Preez NF-d, Ramlagan S, Fomundam H. Use of traditional complementary and alternative medicine for HIV patients in KwaZulu-Natal, South Africa. BMC Public Health. 2008;8(1):1–14. 10.1186/1471-2458-8-255 18652666 PMC2503977

[10] Peltzer K. Utilization and practice of traditional/complementary/alternative medicine (TM/CAM) in South Africa. Afr J Tradit Complement Altern Med. 2009;6(2):175–185. 20209010 PMC2816568

[11] Tangkiatkumjai M, Boardman H, Walker D-M. Potential factors that influence usage of complementary and alternative medicine worldwide: A systematic review. BMC Complement Med Ther. 2020;20(1):1–15. 10.1186/s12906-020-03157-2 33228697 PMC7686746

[12] Masic I. The false science in the biomedicine-a dilemma. Int J Biomed Healthc. 2023;11(1):4–24. 10.5455/ijbh.2023.11.4-24

[13] Alissa EM. Medicinal herbs and therapeutic drugs interactions. Ther Drug Monit. 2014;36(4):413–422. 10.1097/FTD.0000000000000035 24452064

[14] Bordes C, Leguelinel-Blache G, Lavigne J-P, et al. Interactions between antiretroviral therapy and complementary and alternative medicine: A narrative review. Clin Microbiol Infect. 2020;26(9):1161–1170. 10.1016/j.cmi.2020.04.019 32360208

[15] Kretchy IA, Okere HA, Osafo J, Afrane B, Sarkodie J, Debrah P. Perceptions of traditional, complementary and alternative medicine among conventional healthcare practitioners in Accra, Ghana: Implications for integrative healthcare. J Integr Med. 2016;14(5):380–388. 10.1016/S2095-4964(16)60273-X 27641609

[16] Liu L, Tang Y, Baxter GD, Yin H, Tumilty S. Complementary and alternative medicine-practice, attitudes, and knowledge among healthcare professionals in New Zealand: An integrative review. BMC Complement Med Ther. 2021;21(1):1–11. 10.1186/s12906-021-03235-z 33583417 PMC7882070

[17] Nimesh S. The role of pharmacist in the health care system: Current scenario in India. Borneo J Pharm. 2020;3(2):84–89. 10.33084/bjop.v3i2.1325

[18] Dineen-Griffin S, Benrimoj SI, Garcia-Cardenas V. Primary health care policy and vision for community pharmacy and pharmacists in Australia. Pharm Pract. 2020;18(2):1967. 10.18549/PharmPract.2020.2.1967 PMC7243858 32477437

[19] Hussain S, Malik F, Hameed A, et al. Pakistani pharmacy students’ perception about complementary and alternative medicine. Am J Pharmaceut Educ. 2012;76(2):21. 10.5688/ajpe76221 PMC3305930 22438593

[20] Jamshed SQ, Khan MU, Ahmad A, Elkalmi RM. Knowledge, perceptions, and attitudes toward complementary and alternative medicines among pharmacy students of a Malaysian Public University. J Pharm Bioallied Sci. 2016;8(1):34. 10.4103/0975-7406.171686 26957866 PMC4766776

[21] Saha BL, Seam M, Reza O, et al. General perception and self-practice of complementary and alternative medicine (CAM) among undergraduate pharmacy students of Bangladesh. BMC Complement Altern Med. 2017;17(1):1–8. 10.1186/s12906-017-1832-y 28615021 PMC5471669

[22] Truter I. Knowledge and perceptions of pharmacy students towards training in complementary and alternative health care. Health SA Gesondheid. 2005;10(2):3–16. 10.4102/hsag.v10i2.191

[23] Viganò G, Nannei P, Bellavite P. Homeopathy: From tradition to science? J Med Person. 2015;13:7–17. 10.1007/s12682-014-0197-y

[24] Tesch R. Qualitative research: Analysis types and software. Routledge; 2013.

[25] Burke S. Rethinking ‘validity’ and ‘trustworthiness’ in qualitative inquiry: How might we judge the quality of qualitative research in sport and exercise sciences? In: Routledge handbook of qualitative research in sport and exercise. p. 352–362. New York: Routledge; 2016.

[26] Pillay S. A study on the knowledge, attitudes and perceptions of primary health care nurses in the eThekwini Municipality District with regards to the inclusion of homoeopathy in primary health care [Theses and Dissertations]. Durban: Durban University of Technology; 2013.

[27] Naicker S. A survey of medical specialists’ perceptions and interactions with homoeopathy [Theses and Dissertations]. Durban: Durban University of Technology; 2008.

[28] Mann T. A survey to establish perceptions of homoeopathy among pharmacists and pharmacists’ assistants in greater. Johannesburg: University of Johannesburg Repositry; 2010.

[29] Anggriani Y, Ibrahim MIM, Suryawati S, Shafie AA. The impact of Indonesian generic medicine pricing policy on medicine prices. J Generic Med. 2013;10(3–4):219–229. 10.1177/1741134314553605

[30] Nguyen TA, Knight R, Roughead EE, Brooks G, Mant A. Policy options for pharmaceutical pricing and purchasing: Issues for low-and middle-income countries. Health Policy Plann. 2015;30(2):267–280. 10.1093/heapol/czt105 24425694

[31] Ngobese VNB. Experiences of returning patients at a Homoeopathic community clinic. 2018.

[32] Masuku A. Patient satisfaction of homeopathic treatment at a public training university in South Africa [Dissertations and Theses]. Johannesburg: University of Johannesburg Respository; 2022.

[33] Lin LW, Ananthakrishnan A, Teerawattananon Y. Evaluating traditional and complementary medicines: Where do we go from here? Int J Technol Assess Health Care. 2021;37(1):e45. 10.1017/S0266462321000179 33729111

[34] Peter Bai J, Jon W, Amie S, Jon A. Traditional, complementary and alternative medicine use in sub-Saharan Africa: A systematic review. BMJ Global Health. 2018;3(5):e000895. 10.1136/bmjgh-2018-000895 PMC6231111 30483405

[35] Salman Popattia A, Winch S, La Caze A. Ethical responsibilities of pharmacists when selling complementary medicines: A systematic review. Int J Pharm Pract. 2018;26(2):93–103. 10.1111/ijpp.12425 29315916

[36] Jahan F, Al-Ward MM, Siddiqui MA, Al-Khouri MA-J. Medical students knowledge and perception regarding complementary and alternative medicine. J Health Educ Res Dev. 2015;3:1–5.

[37] Kanjanarach T, Krass I, Cumming RG. Australian community pharmacists’ practice in complementary medicines: A structural equation modeling approach. Patient Educ Couns. 2011;83(3):352–359. 10.1016/j.pec.2011.05.003 21621945

[38] Thandar Y, Mosam A, Botha J. Community pharmacists’ knowledge, attitude and practices towards the use of complementary and alternative medicines in Durban, South Africa. Health SA Gesondheid. 2019;24(1):1–6. 10.4102/hsag.v24i0.1029 PMC6917453 31934396

